# Effects of cue modality and emotional category on recognition of nonverbal emotional signals in schizophrenia

**DOI:** 10.1186/s12888-016-0913-7

**Published:** 2016-07-07

**Authors:** Bastian D. Vogel, Carolin Brück, Heike Jacob, Mark Eberle, Dirk Wildgruber

**Affiliations:** Department of Psychiatry and Psychotherapy, University of Tübingen, Calwerstraße 14, Tübingen, 72076 Germany

**Keywords:** Emotion, Schizophrenia, Modality, Alluring, Prosody, Facial expression, Vocal expression

## Abstract

**Background:**

Impaired interpretation of nonverbal emotional cues in patients with schizophrenia has been reported in several studies and a clinical relevance of these deficits for social functioning has been assumed. However, it is unclear to what extent the impairments depend on specific emotions or specific channels of nonverbal communication.

**Methods:**

Here, the effect of cue modality and emotional categories on accuracy of emotion recognition was evaluated in 21 patients with schizophrenia and compared to a healthy control group (*n* = 21). To this end, dynamic stimuli comprising speakers of both genders in three different sensory modalities (auditory, visual and audiovisual) and five emotional categories (happy, alluring, neutral, angry and disgusted) were used.

**Results:**

Patients with schizophrenia were found to be impaired in emotion recognition in comparison to the control group across all stimuli. Considering specific emotions more severe deficits were revealed in the recognition of alluring stimuli and less severe deficits in the recognition of disgusted stimuli as compared to all other emotions.

Regarding cue modality the extent of the impairment in emotional recognition did not significantly differ between auditory and visual cues across all emotional categories. However, patients with schizophrenia showed significantly more severe disturbances for vocal as compared to facial cues when sexual interest is expressed (alluring stimuli), whereas more severe disturbances for facial as compared to vocal cues were observed when happiness or anger is expressed.

**Conclusion:**

Our results confirmed that perceptual impairments can be observed for vocal as well as facial cues conveying various social and emotional connotations. The observed differences in severity of impairments with most severe deficits for alluring expressions might be related to specific difficulties in recognizing the complex social emotional information of interpersonal intentions as compared to “basic” emotional states.

Therefore, future studies evaluating perception of nonverbal cues should consider a broader range of social and emotional signals beyond basic emotions including attitudes and interpersonal intentions. Identifying specific domains of social perception particularly prone for misunderstandings in patients with schizophrenia might allow for a refinement of interventions aiming at improving social functioning.

**Electronic supplementary material:**

The online version of this article (doi:10.1186/s12888-016-0913-7) contains supplementary material, which is available to authorized users.

## Background

Impairments in the perception of nonverbal emotional signals in schizophrenia have been reported in numerous investigations [1–6]. Meta-analyses revealed pronounced deficits in identifying, categorizing and differentiating emotional cues such as facial expressions or speech prosody [7–9]. Research indicates that the respective deficits appear to span a broad range of distinct emotions. However, some differences between specific emotions have also been reported, suggesting greater difficulties in the perception of negative emotions such as fear [10, 11], anger or sadness [6, 12–15] compared to the perception of positive emotions such as joy.

Moreover, the modality of the stimuli might affect the extent of difficulties, since meta-analyses report larger effect sizes for the decoding of prosodic (*Cohen’s d* = -1.24 [9]) as compared to facial cues to emotions (*Cohen’s d* = -.81 [8] and *d* = -.91 [7]).

Considering multimodal cues, information about the emotions of others is usually conveyed via facial and vocal cues simultaneously in everyday life [16], and it has been demonstrated in healthy subjects that audiovisual cues facilitate emotion recognition at the level of higher recognition accuracy as well as faster response-times [17]. However, so far research in patients with schizophrenia has mostly focused on studying perception of unimodal social cues, whereas only very few studies evaluated perception of audiovisual nonverbal emotional signals [18–21].

Moreover, systematic studies directly comparing modality-dependent impairments of emotion recognition in patients with schizophrenia are rare and differ in their results.

Simpson et al. [18] reported that patients with schizophrenia have perceptual deficits to a comparable degree in both unimodal conditions (auditory only: Cohen’s *d* = .88, visual only: Cohen’s *d* = .82), but show less impairment in the audiovisual condition (Cohen’s *d* = .39) and suggested that patients with schizophrenia benefit even more from multimodal stimulus presentation than healthy controls. In contrast, Fiszdon et al. [19] observed that patients with schizophrenia showed perceptual deficits in the auditory only condition (Cohen’s *d* = .68) but even more severe impairments in the audiovisual condition (Cohen’s *d* = 1.03). Therefore these authors, while not evaluating the visual only condition in their study, concluded that patients with schizophrenia benefit less from a multichannel task presentation as compared to healthy controls.

The current study aimed at investigating the ability of patients with schizophrenia to decode emotions from isolated facial, vocal, and combined facial and vocal cues using an approach that allows direct comparison between the different modalities of nonverbal emotional communication. A balanced emotion recognition task was employed to clarify issues concerning the stimulus valences.

Based on the research findings mentioned above, we hypothesized that:

1. Compared to the performance of healthy control subjects, patients with schizophrenia would show decreased accuracy in the recognition of nonverbal emotional cues across all stimulus conditions.
2. Patients with schizophrenia would tend to have more difficulties recognizing negative emotions compared to positive emotions.
3. The severity of impairment would differ among the visual, auditory and audiovisual domain. More specifically, more severe deficits are expected for auditory cues compared to visual cues.

## Methods

### Subjects

Twenty-one patients with a diagnosis of schizophrenia or schizoaffective disorder (SCZ) and twenty-one healthy controls (CON) volunteered to participate in this study. At the time of the study, all patients received treatment at the Department of General Psychiatry and Psychotherapy at the University of Tübingen including antipsychotic medication and psychotherapy. All patients were initially diagnosed according to DSM-IV standards by experienced clinicians, and the diagnosis was confirmed upon entering the study using the Structured Clinical Interview for DSM-IV (SCID, Wittchen H-U, Zaudig, M. & Fydrich, T. [22]). Healthy control participants were recruited from the pool of employees of the Medical Center of the University of Tübingen and from their acquaintances. All controls were selected to match patients in terms of age, gender, IQ and education level. Controls were screened to exclude current or past psychiatric disorders using the Mini-International Neuropsychiatric Interview (M.I.N.I.) [23, 24]. All participants spoke German on the level of a native speaker, had normal or corrected to normal vision and hearing and had a sufficient level of everyday functioning to complete the task employed in this study. The majority of the participants in both groups were students. All of the control group’s participants and fourteen of the patients were either still full time students or employed within the last year prior to participation in the study. In addition to socio-demographic data, we assessed the scores of the Positive and Negative Syndrome Scale (PANSS) [25], as well as the Personal and Social Performance Scale (PSP) [26] and utilized the “Mehrfach-Wortschatz-Intelligenz-Test” (MWT-B) [27] as a measure to approximate IQ. An overview of the assessed data is provided in Table 1.

**Table 1:** Socio-demographic data and symptom assessment of participants

	*Schizophrenia (n = 21)*			*Control (n = 21)*			
	*mean value*	*standard deviation*	*range*	*mean value*	*standard deviation*	*range*	*p-value*
Gender:							
*Male*	13			13			
*Female*	8			8			
IQ	109.81	15.93	89–145	116.90	17.07	94–145	.17
Education (given as school years)	11.33	1.65	9–13	11.52	1.60	9–13	.71
Age	36.52	12.44	20–65	36.29	11.13	22–55	.95
PSP:	75.67	12.95	36–95				
PANSS:							
*Total*	52.52	18.84	30–89				
*Positive*	11.67	5.36	7–27				
*Negative*	12.86	6.73	7–29				
*General*	28.00	9.66	16–44				

*p-values of independent t-tests*

### Stimulus material

The stimulus material comprised 20 videos (audiovisual condition, AV), 20 muted videos (visual only condition, VO) and 20 sound recordings (audio only condition, AO) of four professional actors (2f, 2 m). Each stimulus included the recording of one actor speaking one of four single words, consisting of two syllables. The words were selected and balanced based on the results of a previous assessment of their valence and arousal [17, 28–30] on a 9-point Self-Assessment Manikin scale [31] and had a neutral meaning (Möbel = furniture (female actor), Gabel = fork (male actor); Zimmer = room (male actor), Objekt = object (female actor); mean valence scores ± S.D.: 4.9 ± 0.4). While speaking, the actors expressed one of five emotional connotations – happy, alluring, neutral, angry or disgusted—by means of facial expressions and modulations of the tone of their voice.

These emotional connotations were selected with the aim of creating a balanced task design with respect to the number of emotional categories with positive valence (happy and alluring) and negative valence (disgusted and angry) matched for arousal level. Alluring stimuli were selected as the second category of nonverbal cues with a positive valence due to the relevance in social interaction and the conceptual distinction from happy cues [17, 29, 30, 32, 33], the only positive category within the concept of “basic emotions” according to Ekman and Friesen [34]. During recording of alluring cues the actors were asked to nonverbally communicate sexual interest in an inviting manner. The resulting alluring stimuli were relatively uniform across actors with a soft and sustained intonation in the lower frequency spectrum, slow changing facial expressions, mostly with a slight smile and a slight widening of the palpebral fissure and a lifting of one or both eyebrows.

In total, each of the four words was expressed with each of the five emotional connotations at the nonverbal level resulting in 20 different combinations. Each of these combinations was presented in three different modalities (AO, VO, AV) leading to a total set of 60 stimuli to judge. Regardless of presentation modality, the participants were asked to judge the emotional state of the speakers based on their subjective impression by choosing one of the five different emotional categories included in the study.

The muted videos and sound recordings were produced by separating the respective information (visual or auditory) from the 20 original audiovisual recordings (resolution = 720 × 576 pixels, sound = 48 kHz, 16 bit, M_duration_ = 965 ms, SD = 402). A prestudy yielded a gradual proportion of correct classifications for the final stimulus set: 57 % (AO), 70 % (VO), 86 % (AV). The stimuli used in the present study were a subset of stimuli used in previous studies and were found to be reliable and valid measures of emotion recognition abilities, with emotional information identified well above chance level for each stimulus [17, 32, 33]. Details on production, selection and pre-evaluation of the stimulus material can be found in these studies.

### Experimental design

The visual and audiovisual stimuli were presented on a personal computer equipped with a 17-in. flat screen (LG FLATRON L1953PM with a resolution of 800 × 600 pixels) and headphones (Sennheiser, HD 515). Sound volume was adjusted to comfortable hearing levels individually for each participant. The experiment took place in a quiet room, in which the participants were seated in a comfortable position in front of the computer-screen. Presentation of each stimulus had the following sequence: First, the verbal labels of the five emotional categories to choose from appeared for 1 s on the screen in a horizontal order to remind participants of their answer options. Second, a yellow fixation cross and a pure tone (302 Hz) were presented simultaneously for 1 s to direct the participants’ attention. Third, either a video, muted video or sound recording was presented. Followed by fourth, a second presentation of the answer options and, fifth, a visual feedback (700 ms duration) of the chosen answer. Responses were required within a time period of 10 s time-locked to the onset of the stimulus. The total trial duration varied from 3.7 to 12.7 s depending on the stimulus duration and the required response time. Participants conveyed their decision via a button press on a Cedrus RB-730 response pad. The order of the stimuli was fully randomized regardless of modality.

To avoid effects attributed to the positions of the emotional categories on the screen, the ordering of labels was varied among participants. Permutations included switching the positions of labels for negative (anger, disgust) or positive emotions (happiness, alluring) to different positions on the right or the left side of the screen while the label “neutral” always remained in the center. To become familiar with the experimental setting each participant completed a short training session, comprising 15 trials not included in the main experiment.

### Data analysis

Data analysis focused on the accuracy of patients’ responses as measures of performance. To this end hit rates (= proportion of correct responses) were calculated for each participant. Hit rates were averaged among stimuli pertaining to the same emotional category and cue modality and subjected to a mixed-model design analysis of variances (ANOVA) including modality (AO, VO, AV) and emotional category (happy, alluring, neutral, angry, disgusted) as within-subject factors and group (SCZ, CON) as between-subject factor. Significant effects involving group were further explored using post hoc comparisons (t-tests).

To evaluate emotion-specific effects the difference between the mean value of each emotional category and the average value of the remaining four categories was compared between groups using t-tests.

To clarify if patients have more difficulties with negative emotions, the difference between the hit rates for the two positive and the two negative emotions was taken and compared between the two groups using an independent *t*-test.

A similar approach as described above was used for the reaction times as another performance measure. The reaction times of all trials were averaged among stimuli pertaining to the same emotional category as well as cue modality and subjected to a mixed-model design analysis of variances (ANOVA) with the same parameters as described above. Again significant effects involving group were further explored using post hoc comparisons (t-tests).

A complete overview of signal detection rates and error patterns is given in the Additional file 1. Moreover, group differences greater than 20 % are presented descriptively (see supplement).

Finally, to investigate how demographical and clinical factors correlated with the overall hit rate and the reaction time, an explorative data analysis was performed using the Pearson correlation coefficient.

The data was analyzed using IBM SPSS Statistics 21. Significance levels were set at *p* < .05, Greenhouse-Geisser-corrected.

## Results

### Accuracy rates: ANOVA results

The ANOVA revealed significant main effects for group (*F* (1, 40) = 6.89, *p* = .012), for modality (*F* (1.94, 77.72) = 143.08, *p* < .001) and for emotional category (*F* (3.39, 135.73) = 8.66, *p* < .001). Furthermore the ANOVA indicated significant two-way interactions between emotional category and group (*F* (3.39, 135.73) = 2.76, *p* = .039) and between modality and emotional category (*F* (5.47, 218.74) = 27.58, *p* < .001). Moreover, the three-fold interaction between modality, emotional category and group revealed significant results (*F* (5.47, 218.74) = 3.37, *p* = .005). In the following, the significant effects concerning group are further explored.

### Accuracy rates: Main effect of group and interaction with emotional category

**Fig. 1:**
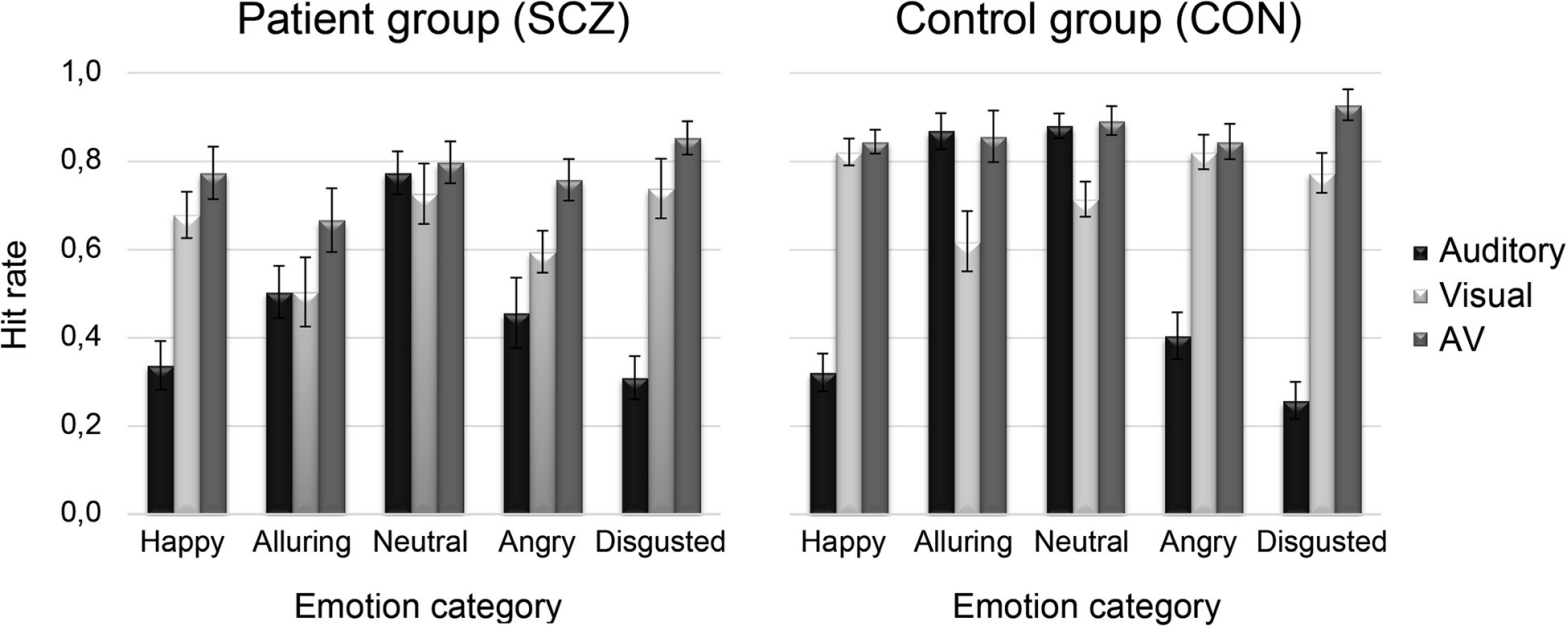
Hit rates of the patients with schizophrenia (*left*) and the control group (*right*) for each emotional category in the different modalities. The bars represent the mean hit rates in the auditory (*black*), the visual (*light gray*) and the audiovisual modality (*gray*). Each error bar visualizes the corresponding standard error

A post hoc *t*-test conducted on mean overall hit rates of both groups shows a significantly reduced overall accuracy across emotional categories in patients with schizophrenia as compared to controls, SCZ: *M* = .63, *SD* = .14; CON: *M* = .72, *SD* = .07; *t* (29.8) = -2.62, *p* = .014. The average hit rates for each emotion and modality are illustrated in Fig. 1.

Further analysis with a post hoc comparison *t*-test for the average hit rates for single emotions as compared to all other emotions yielded a significant group difference for the recognition of alluring stimuli, *t* (40) = - 3.01, *p* = .005, and disgusted stimuli, *t* (40) = 2.25, *p* = .030, indicating more severe impairments for recognition of alluring stimuli and less severe impairments for recognition of disgusted stimuli as compared to the other emotions (see Table 2).

**Table 2:** *Group mean values of the overall hit rate and each single emotion*

	SCZ		CON		Statistics	
	M	SD	M	SD	*p*-value	Cohen’s d
overall	.63	.14	.72	.07	*p* = .014	−.83
happy	.59	.20	.66	.11	*p* = .196	−.44
alluring	.56	.25	.78	.19	*p* = .002	−1.02
neutral	.77	.20	.83	.11	*p* = .215	−.38
angry	.60	.20	.69	.13	*p* = .106	−.55
disgusted	.63	.19	.65	.13	*p* = .698	−.13

*M = Mean value, SD = standard derivation, p-value = p-value of the independent t-test, Cohen’s d = measure of the effect size*

Group mean values (M) of the overall hit rate and the single emotion differences and their standard derivation (SD) are shown in Table 2.

### Accuracy rates: Comparison of negative and positive emotional valence

The recognition accuracies of the two negative (*M* = .62, SD = .16) and the two positive (*M* = .58, SD = .19) emotions did not differ significantly in the patient group, *t* (20) = - .38, *p* = .182.

### Accuracy rates: The effect of cue modality

Both groups had the highest hit rates in the audiovisual modality (SCZ: *M* = .77; CON: *M* = .87), followed by the visual modality (SCZ: *M* = .65; CON: *M* = .75) and the lowest hit rates in the auditory modality (SCZ: *M* = .48; CON: *M* = .55). The ANOVA revealed no significant interaction between group and modality. Nonetheless, the respective effect sizes were calculated (see Table 3) to quantify the observed effects and enable better estimation of necessary sample sizes in future research concerning modality effects.

**Table 3:** *Group mean values of the three modalities*

	SCZ		CON		Statistics	
	M	SD	M	SD	*p*-value	Cohen’s d
auditory	.48	.16	.55	.09	*p* = .091	−.55
visual	.65	.18	.75	.09	*p* = .024	−.72
audiovisual	.77	.15	.87	.10	*p* = .013	−.80

*M = Mean value, SD = standard derivation, p-value = p-value of the independent t-test, Cohen’s d = measure of the effect size*

Furthermore the significant three-fold interaction between modality, emotion and group was evaluated. To this end, the averaged visual only and the averaged auditory only hit rates were compared for each single emotion using a post hoc comparison *t*-test. This analysis revealed significant group differences for the recognition of happy, *t* (40) = - 2.25, *p* = .030, alluring, *t* (40) = 2.09, *p* = .043, and angry stimuli, *t* (40) = - 2.65, *p* = .011, indicating modality dependent impairments for the recognition of happy, alluring and angry stimuli. Patients showed more severe deficits for visual cues expressing happiness or anger and more severe deficits for auditory cues expressing sexual interest (alluring stimuli) as compared to the controls (see Table 4).

**Table 4:** *Differences between visual only and auditory only hit rates of each single emotion*

	SCZ		CON		Statistics	
	M	SD	M	SD	*p*-value	Cohen’s d
overall	.17	.16	.20	.09	*p* = .436	.24
happy	.34	.26	.50	.19	*p* = .030	.71
alluring	.00	.43	−.25	.34	*p* = .043	−.66
neutral	−.05	.32	−.17	.21	*p* = .166	−.45
angry	.14	.38	.42	.29	*p* = .011	.84
disgusted	.43	.32	.51	.27	*p* = .346	.30

*M = Mean value, SD = standard derivation, p-value = p-value of the independent t-test, Cohen’s d = measure of the effect size*

### Reaction time: ANOVA results

The ANOVA revealed significant main effects for group (*F* (1, 40) = 6.89, *p* = .043), for modality (*F* (1.55, 61.99) = 11.77, *p* < .001) and for emotional category (*F* (3.35, 134.16) = 42.37, *p* < .001). Furthermore the ANOVA indicated significant interactions between modality and emotion (*F* (5.47, 239.22) = 27.58, *p* < .001) as well as between modality, emotion category and group (*F* (5.98, 239.22) = 2.85, *p* = .011).

### Reaction time: Main effect of group

**Fig. 2:**
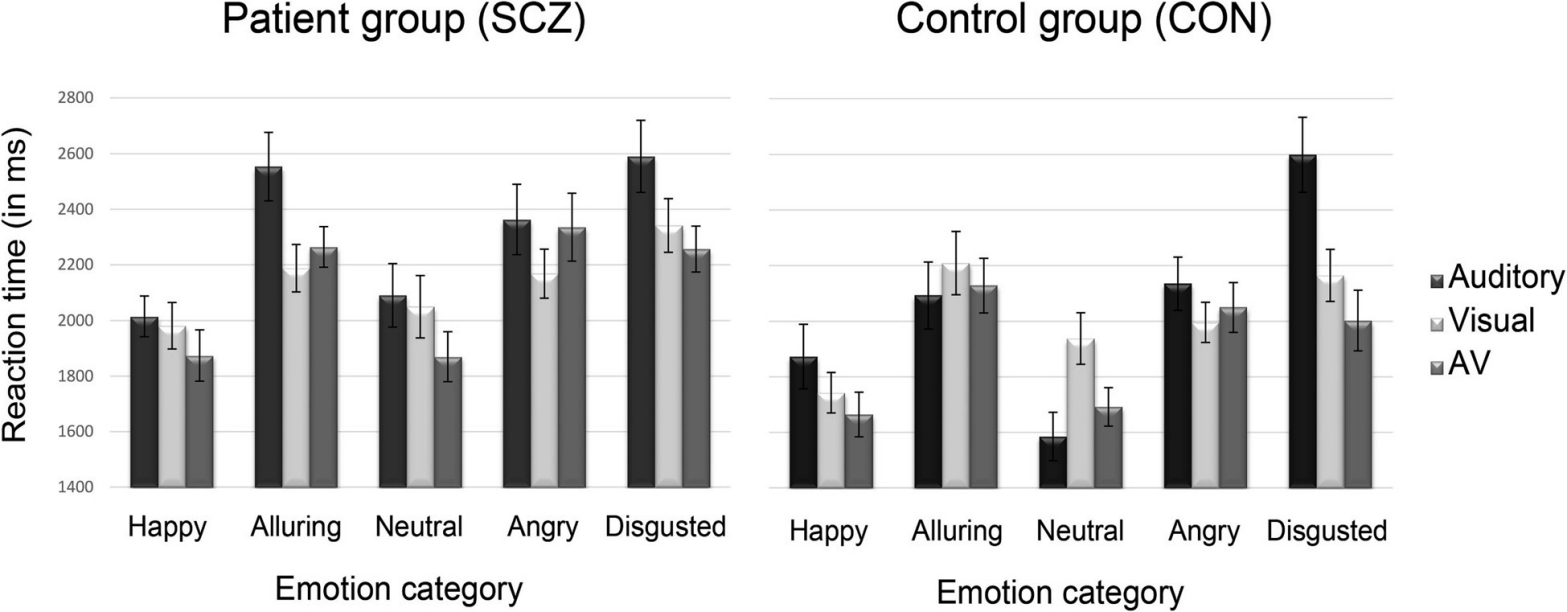
Reaction times of the patients with schizophrenia (*left*) and the control group (*right*) for each emotional category in the different modalities. The bars represent the mean hit rates in the auditory (*black*), the visual (*light gray*) and the audiovisual modality (*gray*). Each error bar visualizes the corresponding standard error

A post hoc *t*-test conducted on reaction times showed a significant increase in reaction time across emotions and modalities in patients with schizophrenia as compared to the control group, SCZ: *M* = 2196 ms, SD = 303 ms; CON: *M* = 1991 ms, SD = 330 ms; *t* (40) = 2.10, *p* = .043. The average reaction times for each emotion and modality are illustrated in Fig. 2.

### Reaction time: interaction of modality, emotion category and group

The post hoc *t*-test revealed an increased reaction time difference between auditory cues and visual cues in the patient group as compared to healthy controls for alluring, *t* (40) = - 3.01, *p* = .004, and neutral stimuli, *t* (25.60) = - 2.24, *p* = .034.

### Correlation with demographical and clinical factors

The overall hit rate correlated in both groups with the years of education (SCZ: *r* = .54, *p* = .012; CON: *r* = .51, *p* = .018) and in the patient group with the total PANSS-score (*r* = -.39, *p* = .025) and the score in the general part of the PANSS (*r* = -.46, *p* = .036). The mean reaction time correlated in the patient group only with the BDI-score (*r* = -.51, *p* = .018) and in the control group with the age (*r* = -.46, *p* = .037) and the years of education (*r* = .57, *p* = .007).

## Discussion

This study investigated emotion recognition in patients with schizophrenia, using dynamic stimuli in auditory, visual, and audiovisual conditions with five different emotional expressions (happy, alluring, neutral, angry and disgusted). Decreased recognition accuracy as well as prolonged reaction time confirm the hypothesized emotion recognition impairments in patients with schizophrenia.

Aiming to improve the analysis of valence effects during perception of nonverbal cues, the number of positive and negative emotional categories was matched in the current study. Moreover, positive and negative cues were matched with respect to their arousal level. Within this study design the hypothesis of greater impairments in recognizing negative emotions as compared to positive emotions was not confirmed. An explanation might be that most previous studies presented only one positive (happiness) and several negative emotions to choose from [35–37]. With the negative emotions probably being more similar to each other, distinguishing one from the other becomes more difficult than distinguishing a single positive one from them. Therefore, in this setup the deficit in emotion recognition could become more obvious with negative valences than with positive valences. The reported valence-effects might thus be the by-product of the task design.

Another possible explanation of the divergence may lie in the selection of specific emotional categories. In contrast to the other categories sexual interest (as expressed in alluring stimuli) is not considered a basic emotion according to the concept of Ekman and Friesen [34]. The recognition of nonverbal cues which convey more complex social and emotional information—such as intentions or attitudes as expressed in alluring cues—might require more or different cognitive resources as the recognition of basic emotions [29, 38–40]. Thus, the absence of a valence effect in the current study might be due to the inclusion of a non-basic positive emotion. More specifically, emotion dependent differences with most severe impairments for alluring expressions might be related to specific difficulties in recognizing complex social emotions or interpersonal intentions as compared to basic emotions.

A striking difference between the recognition of alluring stimuli and the other emotional expressions lays in the modality depending accuracy rates and required reaction times. The patients’ deficits in decoding facial expressions are significantly increased when assessing happy and angry stimuli, which is unexpected because prosodic emotion recognition has been found to be more impaired in most previous studies. This inconsistency might be related to differences in task design. Here, we used only single words for the examination of emotion recognition in prosody and facial expressions whereas previous studies on this topic mainly used full sentences. Single word processing might draw on more basic cognitive resources than processing full sentences and might therefore be less impaired in schizophrenia. The prosodic understanding of patients with schizophrenia, however, seems to be particularly impaired when judging alluring stimuli, which are normally better recognized from prosodic than from visual cues [33]. As alluring stimuli are fundamental for intimate relationships, they are particularly relevant in everyday life. The observed increase in perceptual impairments for these stimuli might indicate that they belong to a subdomain of social perception that is specifically prone for impairments in patients with schizophrenia. To allow for identification and further delineation of such subdomains, a larger variety of emotional categories beyond basic emotions should be evaluated in future research projects [38–40].

Regarding cue modality the emotion recognition impairments did not significantly differ between auditory and visual cues, even though results from former studies indicated such a difference. The effect sizes for the impairments found in this study in decoding prosodic (Cohen’s *d* = -.55) and visual (Cohen’s *d* = -.72) emotional cues were also smaller than effect sizes for prosodic (Cohen’s *d* = -1.24 [9]) and visual (Cohen’s *d* = -.91 [7]) cues reported in meta-analyses. However, the current study represents a direct comparison of impairments across modalities in a single patient group. Indirect comparison of effect sizes observed within separate patient groups that might have differed in severity of symptoms, in contrast, is much more prone to false inference.

Concerning effects sizes of impairments in audiovisual cue perception, our findings are within the range of prior studies [18, 19]. However, the absence of a significant interaction between group and modality in our study neither confirms the assumption of an increase [18] nor a reduction [19] of bimodal facilitation in patients with schizophrenia. Therefore, more research is needed to resolve this issue.

As the PANSS- and the BDI-Score reflect severity of current psychopathological symptoms and can be interpreted as an individual state measure, the results of the correlation analysis suggest that the observed deficits may be state-dependent. This complies with the results of recently published studies [41, 42] which evaluated changes in emotion recognition over time and reported partially state dependent effects and partially trait dependent effects. It should be noted, however, that correlations between emotion recognition impairments and measures of positive or negative psychotic symptoms have been heterogeneous in the literature, ranging from no significant relationship (meta-analytic review of Kohler, Walker et al. [7]) to a correlation with the negative symptoms subscale of the PANSS (review of Chan, Li et al. [8]) as well as other correlations [14, 43, 44]. Hence, a clear relationship between the observed emotion recognition deficit and specific symptoms (e.g. negative/positive) has not been confirmed yet. Since associations between emotion recognition and functional outcome measures have been described in a few prior studies [45–47] these aspects should be systematically evaluated in future research.

### Limitations

Some limitations of our study should be mentioned. First, balancing the task design with respect to the number of positive and negative emotions introduced an imbalance in regard to emotions classified as basic emotions according to Ekman and Friesen [34] that might have influenced the results. Second, it should be mentioned that the male and female actors recorded different words which may lead to a confound between speaker gender and word content. Since all of the words had neutral meanings and the study did not aim to evaluate effects of word content or gender, however, this confound might be considered to have a limited relevance. Third, we did not examine possible effects of medication. Even though a systemic review [48] on this issue showed no substantial improvement in facial affect recognition after treatment with either typical or atypical antipsychotic drugs, the medication could still influence task performance. Fourth, due to the small sample size we did not examine possible differences between different subtypes of the illness, e.g. differences between the paranoid and the catatonic subtype. Fifth, only single words were used as stimulus material. As sentences in real life consist of more than one word and provide more prosodic information, the impairments in emotion recognition during auditory and visual perception of full sentences or even longer sections of a conversation might differ substantially.

## Conclusion

Our findings complement the evidence for impairments in emotional recognition in schizophrenia. Yet, it remains unclear if these impairments are accentuated for negative emotions and if these impairments differ depending on the modality of the stimuli. In our study, modality effects occurred only for some emotions and with different directions, namely more severe deficits for auditory cues in alluring stimuli and more severe deficits for visual cues in angry and happy stimuli. To resolve these issues, further studies should evaluate group effects in larger samples using task designs balanced for emotional valence and stimulus modality.

Moreover, future studies should include a broader range of nonverbal emotional signals beyond basic emotions, including intentions (e.g. comforting, encouraging, inviting, appeasing) and attitudes (e.g. optimistic, benevolent, skeptical, uncertain) [39]. These signals play an important role in social relationships and might therefore be related to the functional outcome of patients with schizophrenia. This may be especially interesting for studies comparing different modalities, since modality specific effects might vary between basic emotions and more complex social information [33, 40].

The impairments in emotional recognition could amplify insecurities and discomfort in social situations and eventually promote social retreat as a part of negative symptoms, which worsens the prognosis of the patients [49, 50]. Therapies like the “Social Cognition and Interaction Training” (SCIT) [51, 52], aiming at improving perception and understanding of emotions, should therefore be further developed, evaluated, and employed to improve the outcome and quality of life of patients with schizophrenia.

## Abbreviations

ANOVA, analysis of variance; AO, audio only; AV, audiovisual; CON, control group; DSM-IV, “Diagnostic and Statistical Manual of Mental Disorders”, fourth edition; IQ, intelligence quotient; M, mean value; PANSS, Positive and Negative Syndrome Scale; PSP, Personal and Social Performance Scale; SCIT, “Social Cognition and Interaction Training”; SCZ, patient group; SD, standard deviation; VO, visual only

## Additional file

Additional file 1: Signal detection analyses: Additional analysis of group specific signal detection rates and error patterns. (DOCX 33 kb)
