# Supplementary material for: Effects of cue modality and emotional category on recognition of nonverbal emotional signals in schizophrenia
Source: BMC Psychiatry. 2016 Jul 7;16:218. doi: 10.1186/s12888-016-0913-7 (PMC4936116; doi:10.1186/s12888-016-0913-7)
Supplement: Additional file 1: — Signal detection analyses: Additional analysis of group specific signal detection rates and error patterns. (DOCX 33 kb) [file 12888_2016_913_MOESM1_ESM.docx]

**Supplementary data: Signal detection analyses**

**Background**

In contrast to previous research in patients with schizophrenia, the current study did not reveal more severe impairments for recognition of emotional cues with a negative valence (anger and disgust) as compared to those with a positive valence (happy and alluring). Aiming to aid the interpretation of this divergence an additional analysis of group specific signal detection rates and error patterns was performed.

**Methods**

For each stimulus category and each modality the given answers were averaged among participants of each group. The results are presented for the overall effects as well as for each modality for each group (Patients = Table 1-4, Controls = Table 5-8). In a second step, the differences between both groups (patients – controls) were calculated and illustrated in Table 9-12, representing the different modalities. Table 9 illustrates the differences across modalities and Tables 10-12 the differences in each of the three modalities (Table 10 = auditory only, Table 11 = visual only, Table 12 = audiovisual). Moreover, group differences greater than 20% are presented descriptively and highlighted in red within each table. However, due to the absence of a priori hypothesis on specific differences in error patterns and the problem of multiple analyses we did not perform a formal statistical analysis for significant differences between both groups.

**Results**

Averaged across all modalities as well as in the auditory only condition, the patients categorized alluring stimuli less frequently as alluring and more often as neutral or happy than healthy controls. In the visual only condition, the patients categorized angry stimuli less frequently as angry and more often as neutral or disgusted than healthy controls. The other categorizing errors were rather similar in both groups.

**Discussion**

Comparing the two groups, the analyses of group specific signal detection rates revealed no signs of increased mistakes or relevant error patterns differences when it comes to categorizing the three basic emotions. Again, a valance effect concerning negative emotions could not be found.

The results gained from the signal detection analyses support the results from the accuracy rates analyses. Again, patients with schizophrenia were found to be impaired in decoding alluring stimuli and again with more severe impairments in the auditory only modality. The more frequent misinterpretation of alluring stimuli as neutral could be a specific problem of the patients when decoding complex social and emotional information. Therefore, future projects should investigate a greater variety of “non-basic” emotional cues.

**Tables**

**Table 1.** *Signal detection rates and error patterns of the patient group averaged across modalities.*

| **Overall** | | **Chosen category** | | | | |
| --- | --- | --- | --- | --- | --- | --- |
|  |  | **Happy** | **Alluring** | **Neutral** | **Angry** | **Disgusted** |
| **Stimulus category** | **Happy** | 0.60 | 0.03 | 0.32 | 0.04 | 0.01 |
|  | **Alluring** | 0.15 | 0.56 | 0.25 | 0.02 | 0.02 |
|  | **Neutral** | 0.02 | 0.06 | 0.77 | 0.12 | 0.03 |
|  | **Angry** | 0.04 | 0.01 | 0.31 | 0.60 | 0.04 |
|  | **Disgusted** | 0.04 | 0.06 | 0.08 | 0.19 | 0.63 |

**Table 2.** *Signal detection rates and error patterns of the patient group for auditory only stimuli.*

| **Auditory only** | | **Chosen category** | | | | |
| --- | --- | --- | --- | --- | --- | --- |
|  |  | **Happy** | **Alluring** | **Neutral** | **Angry** | **Disgusted** |
| **Stimulus category** | **Happy** | 0.34 | 0.02 | 0.52 | 0.12 | 0.00 |
|  | **Alluring** | 0.15 | 0.50 | 0.30 | 0.02 | 0.02 |
|  | **Neutral** | 0.07 | 0.06 | 0.77 | 0.08 | 0.01 |
|  | **Angry** | 0.08 | 0.00 | 0.44 | 0.46 | 0.02 |
|  | **Disgusted** | 0.11 | 0.18 | 0.20 | 0.20 | 0.31 |

**Table 3.** *Signal detection rates and error patterns of the patient group for visual only stimuli.*

| **Visual only** | | **Chosen category** | | | | |
| --- | --- | --- | --- | --- | --- | --- |
|  |  | **Happy** | **Alluring** | **Neutral** | **Angry** | **Disgusted** |
| **Stimulus category** | **Happy** | 0.68 | 0.05 | 0.26 | 0.00 | 0.01 |
|  | **Alluring** | 0.18 | 0.50 | 0.31 | 0.00 | 0.01 |
|  | **Neutral** | 0.00 | 0.05 | 0.73 | 0.18 | 0.05 |
|  | **Angry** | 0.00 | 0.01 | 0.32 | 0.60 | 0.07 |
|  | **Disgusted** | 0.01 | 0.00 | 0.01 | 0.24 | 0.74 |

**Table 4.** *Signal detection rates and error patterns of the patient group for audiovisual stimuli.*

| **Audiovisual** | | **Chosen category** | | | | |
| --- | --- | --- | --- | --- | --- | --- |
|  |  | **Happy** | **Alluring** | **Neutral** | **Angry** | **Disgusted** |
| **Stimulus category** | **Happy** | 0.77 | 0.02 | 0.18 | 0.01 | 0.01 |
|  | **Alluring** | 0.14 | 0.67 | 0.15 | 0.02 | 0.01 |
|  | **Neutral** | 0.00 | 0.07 | 0.80 | 0.11 | 0.02 |
|  | **Angry** | 0.04 | 0.01 | 0.16 | 0.76 | 0.04 |
|  | **Disgusted** | 0.00 | 0.00 | 0.02 | 0.12 | 0.85 |

**Table 5.** *Signal detection rates and error patterns of the control group averaged across modalities.*

| **Overall** | | **Chosen category** | | | | |
| --- | --- | --- | --- | --- | --- | --- |
|  |  | **Happy** | **Alluring** | **Neutral** | **Angry** | **Disgusted** |
| **Stimulus category** | **Happy** | 0.66 | 0.03 | 0.29 | 0.02 | 0.00 |
|  | **Alluring** | 0.09 | 0.78 | 0.12 | 0.00 | 0.00 |
|  | **Neutral** | 0.01 | 0.08 | 0.83 | 0.08 | 0.00 |
|  | **Angry** | 0.05 | 0.02 | 0.23 | 0.69 | 0.02 |
|  | **Disgusted** | 0.00 | 0.12 | 0.08 | 0.14 | 0.65 |

**Table 6.** *Signal detection rates and error patterns of the control group for auditory only stimuli.*

| **Auditory only** | | **Chosen category** | | | | |
| --- | --- | --- | --- | --- | --- | --- |
|  |  | **Happy** | **Alluring** | **Neutral** | **Angry** | **Disgusted** |
| **Stimulus category** | **Happy** | 0.32 | 0.00 | 0.63 | 0.05 | 0.00 |
|  | **Alluring** | 0.04 | 0.87 | 0.10 | 0.00 | 0.00 |
|  | **Neutral** | 0.01 | 0.11 | 0.88 | 0.00 | 0.00 |
|  | **Angry** | 0.13 | 0.02 | 0.43 | 0.40 | 0.01 |
|  | **Disgusted** | 0.01 | 0.37 | 0.19 | 0.17 | 0.26 |

**Table 7.** *Signal detection rates and error patterns of the control group for visual only stimuli.*

| **Visual only** | | **Chosen category** | | | | |
| --- | --- | --- | --- | --- | --- | --- |
|  |  | **Happy** | **Alluring** | **Neutral** | **Angry** | **Disgusted** |
| **Stimulus category** | **Happy** | 0.82 | 0.07 | 0.11 | 0.00 | 0.00 |
|  | **Alluring** | 0.18 | 0.62 | 0.19 | 0.01 | 0.00 |
|  | **Neutral** | 0.01 | 0.08 | 0.71 | 0.18 | 0.01 |
|  | **Angry** | 0.00 | 0.00 | 0.15 | 0.82 | 0.02 |
|  | **Disgusted** | 0.00 | 0.00 | 0.04 | 0.19 | 0.77 |

**Table 8.** *Signal detection rates and error patterns of the control group for audiovisual stimuli.*

| **Audiovisual** | | **Chosen category** | | | | |
| --- | --- | --- | --- | --- | --- | --- |
|  |  | **Happy** | **Alluring** | **Neutral** | **Angry** | **Disgusted** |
| **Stimulus category** | **Happy** | 0.85 | 0.01 | 0.14 | 0.00 | 0.00 |
|  | **Alluring** | 0.06 | 0.86 | 0.08 | 0.00 | 0.00 |
|  | **Neutral** | 0.01 | 0.05 | 0.89 | 0.05 | 0.00 |
|  | **Angry** | 0.01 | 0.02 | 0.11 | 0.85 | 0.01 |
|  | **Disgusted** | 0.00 | 0.00 | 0.00 | 0.07 | 0.93 |

**Table 9.** *Differences of signal detection rates and error patterns between both groups averaged across modalities.*

|  |  | **Chosen category** | | | | |
| --- | --- | --- | --- | --- | --- | --- |
| **Overall** |  | **Happy** | **Alluring** | **Neutral** | **Angry** | **Disgusted** |
| **Stimulus category** | **Happy** | -0.07 | 0.00 | 0.03 | 0.03 | 0.01 |
|  | **Alluring** | 0.06 | -0.22 | 0.13 | 0.01 | 0.02 |
|  | **Neutral** | 0.01 | -0.02 | -0.06 | 0.05 | 0.02 |
|  | **Angry** | -0.01 | -0.01 | 0.08 | -0.09 | 0.03 |
|  | **Disgusted** | 0.04 | -0.06 | 0.01 | 0.04 | -0.02 |

**Table 10.** *Differences of signal detection rates and error patterns between both groups for auditory only stimuli.*

|  |  | **Chosen category** | | | | |
| --- | --- | --- | --- | --- | --- | --- |
| **Auditory only** | | **Happy** | **Alluring** | **Neutral** | **Angry** | **Disgusted** |
| **Stimulus category** | **Happy** | 0.02 | 0.02 | -0.11 | 0.07 | 0.00 |
|  | **Alluring** | 0.11 | -0.37 | 0.21 | 0.02 | 0.02 |
|  | **Neutral** | 0.06 | -0.05 | -0.11 | 0.08 | 0.01 |
|  | **Angry** | -0.05 | -0.02 | 0.01 | 0.05 | 0.01 |
|  | **Disgusted** | 0.10 | -0.19 | 0.01 | 0.04 | 0.05 |

**Table 11.** *Differences of signal detection rates and error patterns between both groups for visual only stimuli.*

| **Visual only** | | **Chosen category** | | | | |
| --- | --- | --- | --- | --- | --- | --- |
|  |  | **Happy** | **Alluring** | **Neutral** | **Angry** | **Disgusted** |
| **Stimulus category** | **Happy** | -0.14 | -0.02 | 0.15 | 0.00 | 0.01 |
|  | **Alluring** | 0.00 | -0.12 | 0.12 | -0.01 | 0.01 |
|  | **Neutral** | -0.01 | -0.04 | 0.01 | 0.00 | 0.04 |
|  | **Angry** | 0.00 | 0.01 | 0.17 | -0.23 | 0.05 |
|  | **Disgusted** | 0.01 | 0.00 | -0.02 | 0.05 | -0.04 |

**Table 12.** *Differences of signal detection rates and error patterns between both groups for audiovisual stimuli.*

| **Audiovisual** | | **Chosen category** | | | | |
| --- | --- | --- | --- | --- | --- | --- |
|  |  | **Happy** | **Alluring** | **Neutral** | **Angry** | **Disgusted** |
| **Stimulus category** | **Happy** | -0.07 | 0.01 | 0.04 | 0.01 | 0.01 |
|  | **Alluring** | 0.08 | -0.19 | 0.07 | 0.02 | 0.01 |
|  | **Neutral** | -0.01 | 0.02 | -0.10 | 0.06 | 0.02 |
|  | **Angry** | 0.02 | -0.01 | 0.05 | -0.09 | 0.02 |
|  | **Disgusted** | 0.00 | 0.00 | 0.02 | 0.05 | -0.08 |
